# Shikonin as a Dietary Phytochemical with Multi-Target Anti-Cancer Activities: From Molecular Mechanisms to Translational Applications

**DOI:** 10.3390/nu17193085

**Published:** 2025-09-28

**Authors:** Chun-Yik Lew, Yi-Teng Tang, Amanda Yee-Jing Lee, Zhi-Jian Chin, Wan-Ling Chang, Ching-Hsein Chen, Soi-Moi Chye

**Affiliations:** 1 School of Health Science, IMU University, No. 126, Jalan Jalil Perkasa 19, Bukit Jalil, Kuala Lumpur 57000, Malaysia; 00000034177@student.imu.edu.my (C.-Y.L.); 00000036022@student.imu.edu.my (Y.-T.T.); 00000037390@student.imu.edu.my (A.Y.-J.L.); 00000038198@student.imu.edu.my (Z.-J.C.); 2 Department of Anesthesiology, Chang Gung Memorial Hospital at Chiayi, No. 8, West Section of Jiapu Road, Puzi City 613016, Taiwan; chjack1975@yahoo.com.tw; 3 Department of Microbiology, Immunology and Biopharmaceuticals, College of Life Sciences, A25-303 Room, Life Sciences Hall, National Chiayi University, No. 300, Syuefu Road, Chiayi City 600355, Taiwan; 4 School of Health Science, Division of Applied Biomedical Science and Biotechnology, IMU University, No. 126, Jalan Jalil Perkasa 19, Bukit Jalil, Kuala Lumpur 57000, Malaysia

**Keywords:** shikonin, dietary phytochemicals, anticancer mechanisms, drug resistance, combination therapy

## Abstract

Shikonin, a dietary naphthoquinone phytochemical from the roots of *Lithospermum erythrorhizon*, has gained attention for its anticancer potential. Preclinical studies show that shikonin regulates multiple programmed cell death pathways, including apoptosis, necroptosis, ferroptosis, and pyroptosis, through mechanisms involving reactive oxygen species (ROS) accumulation, mitochondrial dysfunction, and kinase-mediated signalling. Beyond cytotoxicity, shikonin suppresses metastasis by blocking epithelial–mesenchymal transition (EMT) and downregulating matrix metalloproteinase-2 (MMP-2) and matrix metalloproteinase-9 (MMP-9). It also disrupts tumour metabolism by targeting pyruvate kinase isoform M2 (PKM2) and modulating the Warburg effect. Evidence further indicates that shikonin can enhance the efficacy of chemotherapy, targeted therapy, immunotherapy, and radiotherapy, thereby contributing to the reversal of therapeutic resistance. To address limitations related to solubility and bioavailability, novel formulations such as nanoparticles, liposomes, and derivatives like β,β-dimethylacrylshikonin have been developed, showing improved pharmacological profiles and reduced toxicity in experimental models. Overall, the current literature identifies shikonin as a promising dietary phytochemical with diverse anticancer activities, therapeutic synergy, and formulation advances, while highlighting the need for clinical studies to establish its translational potential.

## 1. Introduction

According to the global cancer statistics in 2022, an estimated 20 million new cancer cases and 9.7 million cancer deaths were reported worldwide [1]. Despite decades of advancement in oncology research, cancer remains a major global health challenge, particularly in advanced or metastatic stages. Conventional treatments, such as surgery, chemotherapy, radiotherapy and immunotherapy, have significantly improved survival outcomes and have reduced mortality rates in cancer patients [2]. However, drug resistance driven by genetic mutations, tumour heterogeneity, and tumour microenvironment (TME) remains a critical barrier that hinders the effectiveness of conventional therapies and compromises long-term treatment outcomes [3]. Besides that, these therapies are often associated with systemic and off-target toxicities, characterised by fatigue, dermatological reactions, hepatotoxicity, cardiotoxicity and nephrotoxicity [4,5]. Therefore, natural compounds, especially dietary phytochemicals, have emerged as promising alternative therapeutic approaches due to their diverse biological activities, lower toxicity and accessibility from medicinal plants [6].

Phytochemicals are bioactive compounds derived from plants, including grains, fruits, vegetables, and herbs, recognised for their beneficial effects on human health. They can be categorised into several major classes, such as carotenoids, polyphenols, naphthoquinones and terpenoids, based on their chemical structures [7]. Since antiquity, plant-derived products have been incorporated into traditional medical systems, such as Traditional Chinese Medicine (TCM) and Ayurveda, serving both preventive and therapeutic purposes [8]. For instance, ginger (*Zingiber officinale*) exhibits thermogenic properties that stimulate blood circulation and has long been used to treat respiratory disorders such as coughs and the common cold [9]. In addition, *Lithospermum erythrorhizon* has been traditionally used to reduce body surface heat, relieve congestion and treat skin diseases, burns and injuries [10]. More recently, numerous studies have elucidated the molecular mechanisms through which phytochemicals regulate physiological processes and promote human health. Among these, bioactive compounds such as resveratrol, derived from grapes, and beta-carotene (β-carotene), found in carrots, exhibit antioxidant and anti-inflammatory effects in the treatment of acute pancreatitis [11,12].

With the growing interest in exploring dietary phytochemicals, shikonin has gained significant attention as a bioactive naphthoquinone pigment with both pharmacological and therapeutic potential, particularly in cancer treatment. It is extracted from the roots of *Lithospermum erythrorhizon* (purple gromwell, Zicao), an edible and medicinal plant long used in TCM and occasionally incorporated into functional foods [13,14]. The roots contain dietary fibre, essential fatty acids, and polyphenolic compounds with antioxidant capacity, supporting its nutritional relevance alongside pharmacological use. Its inclusion in medicinal teas, ointments, and decoctions underscores its dual role as both a therapeutic agent and a dietary phytochemical [15]. This traditional use supports its classification as a dietary phytochemical, reinforcing both its reputation for safety and its promise in long-term chemoprevention and integrative cancer care [15,16]. Recent studies show that shikonin suppresses tumour growth in lung, ovarian, and other cancers through diverse mechanisms, including apoptosis induction, cell cycle arrest, inhibition of metastasis, necroptosis, and disruption of tumour metabolism [17]. Its multi-targeted activity and relatively low systemic toxicity highlight shikonin as a compelling candidate for development as both a preventive agent and an adjunct to conventional cancer therapies [18].

In this review, we aim to provide an overview of the chemical and pharmacological profile of shikonin and its derivatives, along with their anticancer mechanisms in regulating tumour progression. This review also discusses the potential of shikonin and its derivatives in combination therapy and the translational studies relevant to future research and clinical applications in cancer treatment.

## 2. Chemistry and Pharmacokinetics

Shikonin is a naphthoquinone compound found abundantly in the roots of the Boraginaceae family, giving them a red-purple appearance [19]. The chemical structure of shikonin consists of a 1, 4-naphthoquinone core substituted with hydroxyl groups at positions 5 and 8 and a hydroxypentenyl side chain at position 2. Its molecular formula is C_16_H_16_O_5_, with a molecular weight of 288.29 g/mol [10]. Shikonin has poor aqueous solubility but is highly soluble in organic solvents such as ethanol and chloroform [14]. Besides that, shikonin is sensitive to light and heat, and it can be easily degraded when exposed to temperatures above 60 °C [20]. Shikonin also exhibits redox activity, which involves the transfer of two protons and two electrons at the naphthazarin moiety [21]. This property enables shikonin to function as a pro-oxidant agent by inducing oxidative stress in cancer cells to suppress tumour growth [22].

### 2.1. Methods of Isolation and Production of Shikonin

Shikonin is commonly isolated using maceration or Soxhlet extraction techniques with organic solvents like ethanol, methanol, hexane, ethyl acetate or chloroform [23,24]. Sagratini et al. reported that Soxhlet extraction using ethyl acetate for 6 h produced the highest yield of alkannin/shikonin mixture (80%) from the roots of *Onosma echioides* [23]. Ethanol is often preferred due to low toxicity, ease of removal and high extraction yield. However, these methods have disadvantages such as long processing times, high energy consumption, risk of compound degradation and low extraction efficiency [25]. To overcome these drawbacks, alternative extraction techniques have been developed. Ultrasonic-assisted extraction (UAE) applies mechanical, thermal and cavitation effects to disrupt cell walls and enhance the release of bioactive compounds [26]. Huang et al. reported the maximum yield of shikonin (1.26%) was extracted using the UAE procedure under optimal conditions of 39 °C with 95% ethanol and 93 W ultrasound power for 87 min [20]. Furthermore, microwave-assisted extraction (MAE) is another effective extraction method, which involves the use of electromagnetic waves to heat the solvent and plant matrix within a closed system [27]. The optimal extraction conditions for shikonin using the MAE technique are 330 W of microwave power with 80% methanol for 12 min [28]. Supercritical carbon dioxide (SC-CO_2_) extraction uses CO_2_ above its critical temperature and pressure to dissolve target compounds from plant material [29]. Shen et al. optimised the conditions of SC-CO_2_ extraction of shikonin at 3 MPa and 40 °C, with a CO_2_ flow rate of 27 L/h for 2 h [30]. These advanced extraction technologies are widely used, particularly in pharmaceutical and food industries, for their high extraction yield and purity, eco-friendly nature and short operation time [31,32].

Following the isolation of shikonin from plants, purification is essential to remove impurities and residual organic solvents. Common separation techniques include high-performance liquid chromatography (HPLC), high-speed counter-current chromatography (HSCCC) and column chromatography [23,33]. Besides that, plant cell culture technique has been employed to produce large quantities of shikonin. For instance, callus culture of *Onosma bulbotrichum* in Murashige and Skoog (MS) medium supplemented with growth hormones yielded 2.6 times more shikonin than wild plant roots [34]. Another study conducted by Yazaki et al. demonstrated that the hairy root culture of *Lithospermum erythrorhizon* infected with *Agrobacterium rhizogenes* produced a similar amount of shikonin as the cell suspension culture [35]. In addition, multiple studies have explored chemical synthesis pathways to generate shikonin and its derivatives [36,37]. Wang et al. developed a total synthesis pathway, which involves the asymmetrical hydrogenation of the Ru (II) catalyst followed by the removal of methyl groups, and results in a high yield (47%) of shikonin production [38].

### 2.2. Shikonin Derivatives and Their Anticancer Activities

Apart from natural extraction, shikonin derivatives are obtained via semi-synthetic routes by chemically modifying the side chain attached to the naphthazarin structure. For instance, Zhou et al. demonstrated the methylation of the phenolic hydroxyl group of shikonin produced its derivative, 5, 8-*O*-dimethylacrylshikonin. This study reported that 5, 8-*O*-dimethyl acylshikonin inhibited the tumour growth in Kunming (KM) mice with subcutaneous injection of Sarcoma S-180 cells [39]. Another research study conducted by Huang et al. found that shikonin oxime derivatives incorporating a sulphur atom exhibited cytotoxic effects against breast cancer, leukaemia and prostate cancer cells [40]. Other derivatives, including α-methylbutyrylshikonin and β, β-dimethylacrylshikonin, exhibited potent cytotoxicity against melanoma, leukaemia, colorectal cancer, gastric cancer, and medullary thyroid carcinoma cells [41,42,43,44,45]. In addition, acetylshikonin produced via acetylation of shikonin showed antitumour effects on oral squamous cell carcinoma (OSCC), non-small cell lung cancer (NSCLC) and colorectal cancer [46,47,48]. Other examples of shikonin derivatives, such as β-hydroxyisovalerylshikonin, deoxyshikonin and isobutyrylshikonin, demonstrated various anticancer activities on pancreatic cancer, cervical cancer and oral cancer [49,50,51,52]. Table 1 summarises the chemical structures, modifications and the anticancer effects of various shikonin derivatives. These findings highlight that the structural modifications of shikonin can significantly improve its pharmacological effects, facilitating the development of novel shikonin-based anticancer drugs.

### 2.3. Pharmacokinetics of Shikonin and Its Pharmaceutical Formulations

The pharmacokinetics of shikonin has been primarily explored using in vivo animal models, such as rats, mice and dogs. Shikonin exhibits relatively low bioavailability due to its poor aqueous solubility and high metabolism rate. For example, a study on the oral administration of Zi-Cao-Cheng-Qi decoction (25 g/kg) in rats reported a blood concentration of shikonin at 0.5 h post-administration of 0.48 µg/mL, with a total plasma protein binding rate of 64.6% [53]. However, another study reported that shikonin was undetectable in the plasma of Sprague Dawley (SD) rats 10 h after an intravenous (IV) injection of shikonin at a dose of 1.5 mg/kg [54]. This evidence suggests that shikonin undergoes a substantial first-pass effect (FPE) and rapid clearance in rats. After that, shikonin undergoes two phases of metabolism, involving the hydroxylation of the naphthoquinone core and glucuronic acid conjugation [55]. Huang et al. found that shikonin upregulated the expression of drug-metabolising enzymes and transporters in primary rat hepatocytes, thereby modulating the detoxification and elimination pathways [56]. Finally, shikonin is excreted via both bile and urine [57]. 

Shikonin has been incorporated into various pharmaceutical formulations to improve its stability, bioavailability and therapeutic potential. A liposome is a spherical lipid-based drug vehicle that exhibits excellent permeability and retention effects, which allow the drug to accumulate in target tissues and promote drug efficacy [58]. For instance, shikonin-loaded liposomes modified with RGD peptide promoted intracellular uptake and enhanced cytotoxicity in cancer cells due to the specific binding between RGD and α_v_β_3_ receptors on the tumour cell surface [59]. Moreover, Guo et al. found that Zinc-Shikonin-Polyethylene Glycol (Zn-SHK-PEG) nanoparticles showed long retention time in the lung, kidney, liver and spleen without systemic toxicity. Zn-SHK-PEG showed significant inhibition of intracellular ROS generation and strong anti-inflammatory effects in a sepsis model [60]. Besides that, nanogel is a type of hydrogel widely used in drug delivery due to its strong water absorption, high drug-loading capacity and long circulation half-life [61]. Sarcoma-targeting-peptide (STP)-modified nanogel increased the delivery of shikonin in osteosarcoma cells and inhibited tumour progression and metastasis through necroptosis. Treatment with shikonin-loaded STP nanogel exhibited high antitumor activity with low systemic toxicity compared to free shikonin [62].

## 3. Mechanisms of Anticancer Action

### 3.1. Induction of Apoptosis by Shikonin

#### 3.1.1. Shikonin-Induced Intrinsic Apoptosis via MAPK Signalling and ROS-Mediated ER Stress

The intrinsic apoptotic pathway is primarily regulated by mitochondrial events and is characterised by the activation of caspases, the key executioners of apoptosis. Caspases such as caspase-3, -7, and -9 degrade cytoskeletal proteins, activate deoxyribonucleases (DNases), and cleave regulatory proteins such as poly (ADP-ribose) polymerase (PARP), thereby preventing DNA repair and committing cells to programmed cell death (PCD) [63,64,65]. Numerous studies have demonstrated that shikonin triggers intrinsic apoptosis through activation of the mitogen-activated protein kinase (MAPK) cascade, leading to downstream caspase-dependent apoptosis [66,67,68,69,70,71].

Shikonin-induced activation of MAPK pathways has been observed in multiple cancer types, including colon carcinoma, chondrosarcoma, blood cancers, melanoma, and renal cancer [66,67,68,69,71,72,73,74,75]. In colon carcinoma, shikonin treatment activated c-Jun N-terminal kinase (JNK) and promoted galectin-1 dimer formation to induce apoptosis in SW-620 cells, whereas radiation-resistant SNU-C5RR cells exhibited apoptosis associated with phosphorylation of JNK, p38, and extracellular signal-regulated kinase (ERK) [67,73]. In colorectal cancer (CRC) HCT-116 and HCT-15 cells, 1.5 µM concentration of shikonin effectively activated the PERK/CHOP and IRE1α/JNK apoptotic pathways, indicating its involvement in oestrogen stress responses [76].

In bone cancer research, acetylshikonin and cyclopropylshikonin dose-dependently (0.5, 2.5, 5, or 10 µM) increased phosphorylation of ERK, JNK, and p38 in Ca-78 and SW-1353 chondrosarcoma cells, while shikonin downregulated ERK activity to induce apoptosis in U2OS osteosarcoma cells [68,75]. Similarly, in blood cancers, shikonin enhanced JNK activation and triggered intrinsic apoptosis in primary effusion lymphoma via ROS generation and significantly elevated p38 and JNK phosphorylation in NB4 cells [72,77]. In melanoma A375M cells, apoptosis was associated with MAPK activation through a 2- to 7-fold increase in JNK and p38 phosphorylation in a dose-dependent manner (0, 2, or 4 µM) [71]. In renal cancer, shikonin was also found to induce apoptosis through downregulating p44/42 MAPK protein in Caki-2 and A-498 cells, but shikonin induces apoptosis by enhancing p38 phosphorylation in Caki-1 and ACHN cells [69,74]. Other cancer models, including triple-negative breast cancer (TNBC) MDA-MB-231 cells, lung adenocarcinoma A546 cells, and pancreatic cancer PANC-1 cells, also demonstrated ERK inhibition and apoptosis induction following shikonin treatment [75]. In summary, shikonin-induced activation of the MAPK-mediated intrinsic apoptotic pathway exhibits pronounced potency in CRC cell lines.

**Figure 1:**
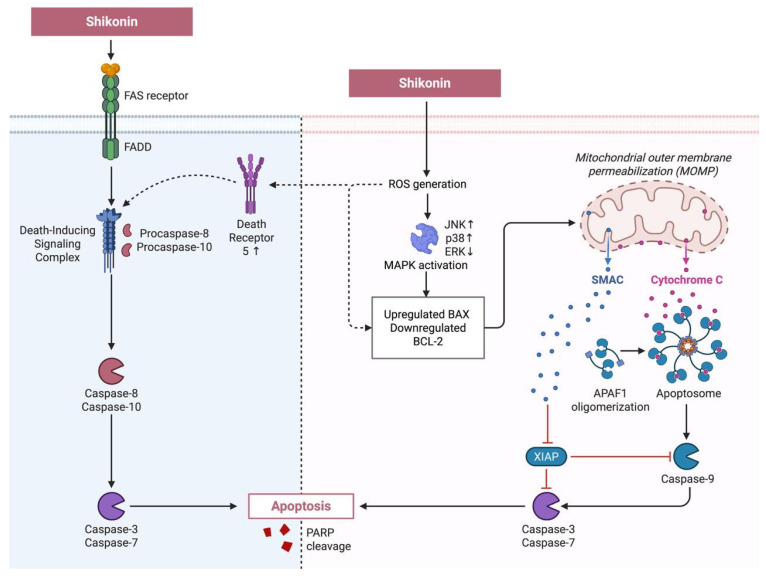
Shikonin induce intrinsic and extrinsic apoptotic pathways in various types of cancer. Upregulated genes (↑) and downregulated genes (↓) are indicated.

Furthermore, shikonin has been found to induce ROS generation and activate apoptotic proteins, resulting in activation of caspase-dependent apoptosis in blood cancer, epidermoid carcinoma, brain cancer, and oral cancer [70,78]. In adult T-cell leukaemia/lymphoma ED- and TL-Om1 cells, shikonin rapidly upregulated ER stress proteins, including activating transcription factor 4 (ATF4), X-box binding protein 1 (XBP-1), and p38 MAPK, leading to apoptosis [70]. In glioma Hs683 cells, shikonin induced apoptosis via increased caspase-3, PERK, and CHOP expression, indicating ER stress-mediated cell death [78]. Similarly, shikonin increased the late apoptosis percentage from 0.82% to 7.23% in oral cancer EC9706 cells by increasing Bax, cleaved caspase-3, and cleaved PARP levels, confirming caspase-dependent apoptosis [79]. Collectively, these findings demonstrate that shikonin effectively triggers intrinsic apoptotic pathways through MAPK activation, ROS generation, and ER stress signalling (Figure 1).

#### 3.1.2. Extrinsic Apoptotic Pathways Triggered by Shikonin: Death Receptor and Caspase-8 Activation

In contrast to the intrinsic apoptotic pathway, the extrinsic apoptotic pathway induces p53-independent apoptosis through the activation of Apo2 ligand/tumour necrosis factor-related apoptosis-inducing ligand (rhApo2L/TRAIL) and Apomab in cancer therapy. These ligands bind to death receptors (DR), such as DR4 and DR5, triggering the death-inducing signalling complex (DISC) by recruiting the adapter Fas-associated death domain (FADD) and procaspases -8 and -10, ultimately leading to caspase-dependent apoptosis [80,81]. Shikonin has been shown to engage this extrinsic apoptotic machinery in multiple cancer models. In GTO primary effusion lymphoma cells, shikonin induced caspase-8-mediated apoptosis, accompanied by increased expression of caspases-3, -8, and -9 [72]. In QBC939 oral cancer cholangiocarcinoma cells, shikonin treatment enhanced cleaved caspase-3 and -8 expression [82]. Similarly, in another cholangiocarcinoma cell line, shikonin also upregulated DR5 and caspases-3, -8, and -9 through ROS-mediated activation of the JNK signalling cascade [22]. In ovarian cancer cell lines, including KURAMOCHI, OVSAHO, CP70, and ascites-derived E04 cells, shikonin activated Fas ligand (FasL)/caspase-8 signalling, as evidenced by increased FasL and cleaved caspase-8, -3, and -7 levels to induce extrinsic apoptosis [83]. These findings suggest that shikonin is capable of triggering extrinsic apoptotic pathways, particularly in ovarian and cholangiocarcinoma models, through death receptor activation and caspase-8 signalling (Figure 1). 

#### 3.1.3. Induction of Apoptosis Through Unique Signalling Pathways

Shikonin has been reported to target specific signalling pathways to induce caspase-dependent apoptosis in a variety of cancer cell lines. In liver cancer, shikonin dose-dependently targets pyrroline-5-carboxylate reductase 1 (PYCR1), leading to suppression of the PI3K/Akt/mTOR signalling pathway in hepatocellular carcinoma (HCC) SNU-449 and Hep-3B cells. Western blot analyses revealed reduced phosphorylation of PI3K, Akt, and mTOR, accompanied by increased levels of cleaved caspase-3, -9, and PARP, indicating that shikonin activates the caspase cascade through inhibition of PI3K/Akt/mTOR signalling [84]. Similarly, in SMMC-7721 HCC cells, shikonin treatment resulted in upregulation of p53 expression and downregulation of phosphorylated Akt and PI3K [85]. Another study reported that shikonin significantly reduced the protein expression of pyruvate kinase M2 (PKM2), HIF-1α, and PHD3, as well as the nuclear translocation of PKM2 and HIF-1α in SMMC-7721 cells [86]. In oral cancer, shikonin inhibited the Akt/mTOR signalling pathway, inducing apoptosis in TE-1 oesophageal cancer cells [87]. In lung cancer, shikonin triggered apoptosis in NSCLC A549 and PC-9 cell lines via the p53/miR-628-3p signalling axis, while in H1299 cells, apoptosis was mediated by downregulation of the survivin signalling pathway [88,89]. In ovarian cancer, shikonin induced apoptosis in SKOV3 and A2780 cell lines by inhibiting the oestrogen signalling pathway and downregulating the expression of its downstream gene, G protein-coupled oestrogen receptor (GPER) [90]. In skin cancer, shikonin inhibited STAT3 signalling, leading to apoptosis in A375 and A2058 melanoma cells [91]. In colon cancer, shikonin induced apoptosis by inhibiting the IL-6/STAT3 signalling pathway in HCT116 and SW480 cells, supported by the downregulation of JAK1 and JAK2 proteins after treatment [92]. Overall, these studies demonstrate that shikonin induces apoptosis across multiple cancer types by targeting unique oncogenic signalling pathways, including PI3K/Akt/mTOR, STAT3, oestrogen, and survivin pathways, ultimately converging on caspase-dependent cell death.

#### 3.1.4. Shikonin and Its Derivatives Induce Apoptosis in Drug-Resistant Cancer Cells

Shikonin also demonstrated incredible antitumor effects in various drug-resistant cancer cell lines. In the epidermal growth factor receptor (EGFR)-T790M-mutant drug-resistant NSCLC cell line H1975, shikonin treatment induced caspase-dependent apoptosis [93]. Similarly, in EGFR-mutated NSCLC H1299 cells, the shikonin derivative E5 inhibited the nuclear translocation of PKM2, thereby suppressing transcriptional activation of oncogenes and triggering apoptosis [94]. In colon cancer, shikonin induced apoptosis in oxaliplatin (OXA)-resistant HCT116 colorectal cancer cells through a ROS-mediated endoplasmic reticulum (ER) stress pathway [95].

Additionally, in 5-fluorouracil (5-FU)-resistant colorectal cancer SNU-C5/5-FUR cells, shikonin regulated glucose-regulated protein 78 (GRP78) and C/EBP homologous protein (CHOP) expression, increased ROS generation, elevated mitochondrial calcium (Ca^2+^) levels about 1.5-fold, and ultimately induced apoptosis [96]. Moreover, the shikonin ester derivative 4-aminophenoacetic acid induced apoptosis in Kirsten rat sarcoma virus (KRAS)-mutant HCT116 colon cancer cells by deactivating the Akt signalling pathway [97]. Cumulatively, these findings highlight that shikonin and its derivatives exert significant antitumor activity against drug-resistant cancers, including NSCLC and colorectal cancer, primarily through apoptosis induction mediated by diverse molecular mechanisms.

#### 3.1.5. Apoptosis Effects of Shikonin Derivatives

Shikonin derivatives have also demonstrated potent antitumor effects by activating both intrinsic and extrinsic apoptotic pathways in various cancer cell lines (Table 2) [68,98,99,100]. In blood cancer, acylshikonin induced apoptosis in leukaemia K562 cells via the intrinsic pathway, as evidenced by increased levels of cleaved caspase-3, -9 and cleaved PARP [101]. In ovarian cancer, β-hydroxyisovaleryl-shikonin induced apoptosis in HeLa cells through inhibition of the PI3K/AKT/mTOR signalling pathway [50]. In colorectal cancer, the semi-synthetic derivative M12 enhanced ROS generation and disrupted mitochondrial membrane potential (MMP), resulting in intrinsic apoptosis, while β, β-dimethylacrylshikonin induced apoptosis in HCT-116 cells by downregulating B-cell lymphoma (Bcl)-2 and Bcl-xL and upregulating Bcl-2-associated X protein (Bax) and BH3-interacting domain death agonist (Bid) [43,50]. In skin cancer, cyclopropylacetylshikonin triggered apoptosis in WM9 and WM164 melanoma cells through activation of the caspase-3/7 cascade, whereas β, β-dimethylacrylshikonin induced NOXA-mediated, caspase-3-dependent apoptosis in BRAF- and NRAS-mutated melanoma subtypes [98,102]. In bone cancer, both acetylshikonin and cyclopropylshikonin upregulated caspase-7, -9, pro-apoptotic NOXA, and the DNA damage marker γH2AX within 24 h of treatment in chondrosarcoma Cal78 and SW-1353 cells [68]. Furthermore, β, β-dimethylacrylshikonin effectively induced apoptosis in chordoma MUG-Chor1 and U-CH2 cell lines through upregulation of pro-apoptotic genes, including NOXA and p53 upregulated modulator of apoptosis (PUMA) [103]. In TNBC MDA-MB-231 cells, treatment with the shikonin derivative E2 modulated the pyruvate dehydrogenase kinase 1 (PDK1) and PDHC/PDK axis, leading to ROS accumulation, upregulation of Bax and Fas proteins, and subsequent apoptosis [99]. In essence, shikonin derivatives demonstrated strong pro-apoptotic activity across multiple cancer types, with particularly notable efficacy in colorectal, bone, skin, and chordoma subtypes, while β, β-dimethylacrylshikonin showed broad-spectrum antitumor activity.

### 3.2. Cell Cycle Arrest

Cell cycle regulation is fundamental for maintaining controlled cell proliferation, and its dysregulation is a hallmark of tumorigenesis [104,105]. The cell cycle comprises the G1, S, G2, and M phases, which are primarily regulated by the cyclin-dependent kinase (CDK) family and controlled by restriction points and checkpoints [106,107]. In cancer therapy, induction of cell cycle arrest represents an important protective mechanism, as it allows cancer cells to respond to oxidative stress and DNA damage, ultimately leading to regulated cell death [104,105,106,107]. Several studies have demonstrated that shikonin induces cell cycle arrest concomitant with regulated cell death pathways in prostate cancer, renal cell carcinoma, osteosarcoma, melanoma, and other cancers [91,108,109,110,111].

Many findings have reported that shikonin and its derivatives induce cell cycle arrest in the sub-G1 and G2/M phases in various cancers. For example, in melanoma A375 and A2058 cell lines, shikonin dose-dependently inhibited cell proliferation and increased cell accumulation in the sub-G1 and G2/M phases. Treatment of melanoma B16F10 cells with the derivative 2-methylbutyryl shikonin also induced G2/M phase arrest, accompanied by reduced cyclin B1 and Cdk1 expression and increased p21 levels [91,111]. Another example of a shikonin derivative, acetylshikonin, induced sub-G1 arrest in osteosarcoma U2OS cells, increasing the sub-G1 population from 5.4% to 16.8% [110]. In prostate cancer models, shikonin exposure increased the G2/M phase population in both parental PC3 and docetaxel-resistant DU145 cells, accompanied by decreased levels of checkpoint proteins cyclin A, cyclin B, and CDK1 [108]. In A549 lung cancer and PANC-1 pancreatic cancer cells, shikonin time-dependently promoted G2/M arrest, while shikonin and cyclopropylshikonin induced G2/M arrest in human chondrosarcoma cells via suppression of Cdc25C expression [68,75].

**Figure 2:**
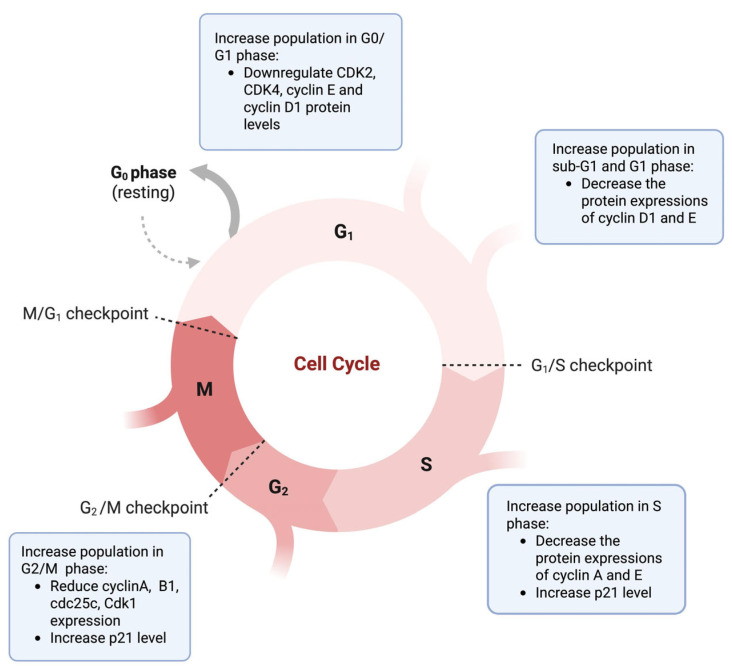
Shikonin induces cell cycle arrest pathways in various cancer.

On the other hand, shikonin can also induce cell cycle arrest at the G1 and S phases. In NSCLC H1299 cells, shikonin dose-dependently increased the cell population in the G0/G1 phase, which was associated with downregulation of CDK2, CDK4, cyclin E, and cyclin D1 protein levels [89]. Shikonin also induced cell cycle arrest at the G1 phase in leukaemia NB4 cells [77]. Additionally, acetylshikonin induced S-phase arrest in leukaemia K562 cells via upregulation of the CDK inhibitor p21 [101], while β-hydroxyisovaleryl-shikonin treatment increased the proportion of ovarian cancer HeLa cells in the S phase [50]. In colorectal cancer, β, β-dimethylacrylshikonin induced G0/G1 arrest in HCT-116 cells [43]. Together, these findings indicate that shikonin and its derivatives can induce cell cycle arrest at multiple phases, including G1, S, G2/M, and sub-G1, across diverse cancer types, supporting their potential as modulators of cell cycle progression in cancer therapy (Figure 2).

### 3.3. Suppression of Metastasis and Invasion

Tumour metastasis remains the primary cause of cancer-related mortality and is a critical determinant of cancer prognosis. Aberrant epithelial–mesenchymal transition (EMT) is one of the hallmarks of metastasis and is commonly associated with invasion due to the development of motility-invasive properties [112]. EMT is characterised by the loss of epithelial phenotypes and the acquisition of mesenchymal cell properties [113]. EMT is a multistep process regulated via various mechanisms such as the expression of EMT-translational factors such as SNAIL, which upregulates the mesenchymal gene expression and downregulates the epithelial gene expression [112,113]. Subsequently, this can result in the “cadherin switch”, in which expression of the epithelial adhesion molecule E-cadherin is reduced while the mesenchymal marker N-cadherin is increased, promoting cell motility and proliferation [112]. Additionally, matrix metalloproteinase-2 (MMP-2) and matrix metalloproteinase-9 (MMP-9) are commonly upregulated during EMT. These enzymes degrade the extracellular matrix, facilitating cell detachment from the primary tumour site, and act as angiogenesis modulators by activating integrin signalling and promoting angiogenic factors such as angiostatin and endostatin [114].

Shikonin has demonstrated the ability to inhibit multiple steps of the metastatic cascade through various signalling pathways. Liu et al. reported that shikonin treatment (2.5 and 5.0 µM) suppressed cell proliferation and induced dose-dependent apoptosis in QBC939 cholangiocarcinoma cells. Cell invasion was reduced, and apoptosis and necrosis were increased, as assessed by Annexin V/propidium iodide flow cytometry [82]. Chen et al. showed that shikonin inhibited metastasis in prostate cancer PC-3 and DU145 cells by downregulating MMP-9 and MMP-2 expression, as observed via gelatine zymography. This effect was mediated by shikonin-induced ROS generation, which activated ERK1/2 and subsequently suppressed MMP-9 and MMP-2 expression [115]. Bhat et al. observed similar effects in shikonin-treated melanoma cells, alongside attenuated EMT via the regulation of the 1-κB⍺/NFκB signalling pathways. I kappaB alpha (1-κB⍺) acts as an inhibitor of nuclear factor kappa B (NF-κB), a factor promoting tumour angiogenesis and metastasis [111].

Huang et al. reported that shikonin, when administered at a dose of 5.0 µM, downregulated tumour necrosis factor receptor-associated protein 1 (TRAP1) expression, resulting in decreased TE-1 human oesophageal cancer cell viability as observed in cell viability assays [87]. Consistent with previous studies, the shikonin-treated TE-1 cells were also found to have decreased MMP2 and MMP9 expression, consequently inhibiting cell migration and proliferation [82,87,115]. Zeng et al. demonstrated in an in vitro pancreatic cell model that β-hydroxyisovaleryl-shikonin was able to suppress EMT progression via inhibiting the phosphorylation of the PI3K/Akt pathway and reducing the expression of N-cadherin, MMP-9, and MMP-2 [49,116]. Similarly, Zhang et al. reported similar effects in shikonin-treated nasopharyngeal carcinoma cells with suppressed cell proliferation and migration [116]. Bao et al. reported findings that shikonin was able to exert antimetastatic properties on TNBC cells as demonstrated on wound healing and Transwell invasion assays. Western blots revealed that shikonin downregulated mesenchymal markers N-cadherin and vimentin expression and upregulated epithelial E-cadherin expression in the treated cells [113]. This was also observed by Mo et al. in human bladder cancer cells, linking EMT suppression with the inhibition of proton efflux pump sodium–hydrogen exchanger 1 (NHE1) by shikonin, though the linking mechanism between shikonin’s inhibitory effects of NHE1 and EMT suppression has yet to be further elucidated [117].

Shikonin’s involvement in EMT inhibition occurs not only with protein signalling pathways but also in gene expression of tumour cells. For instance, it suppresses gene miR-15-5p expression, a microRNA found to promote breast cancer cell EMT and inhibit the expression of tumour suppressor gene phosphatase and tensin homologue (PTEN) [113]. Tabari et al. reported that co-treatment with shikonin and metformin upregulated PTEN expression in MCF-7 breast cancer cells, completely inhibiting cell migration within 24 h at 3.0 µM shikonin and 7.0 mM metformin. Real-time polymerase chain reaction (RT-PCR) analysis revealed decreased expression of pro-EMT genes, such as SNAIL, and increased expression of the anti-EMT gene CDH1 (E-cadherin) [118]. Zhang et al. also elucidated that shikonin suppressed tumour cell metastasis via the p53/miR-361-5p/ZEB1 axis in glioblastoma cells. Western blot studies showed how shikonin upregulates the expression of p53, a mi3-361-5p promoter, which results in miR-365-5p inhibiting ZEB1 overexpression [119]. ZEB1 transcription factors play a major role in promoting EMT by inhibiting E-cadherin and CDH1 expression by binding to their promoters, histone deacetylase (HDAC)1 and HDAC2 [120]. In summary, shikonin exerts anti-metastatic effects by interfering with multiple EMT regulatory mechanisms. It downregulates pro-EMT transcription factors (e.g., SNAIL) and microRNAs (e.g., miR-15-5p), while upregulating tumour suppressors and anti-EMT genes (e.g., CDH1 and PTEN). These changes suppress mesenchymal markers (N-cadherin, vimentin) and enhance epithelial markers (E-cadherin). In parallel, shikonin inhibits MMP expression through modulation of PI3K/Akt and activation of ERK1/2 signalling. Together, these molecular events translate into reduced migration, invasion, and metastatic potential in multiple tumour cell types.

### 3.4. Induction of Necroptosis

**Figure 3:**
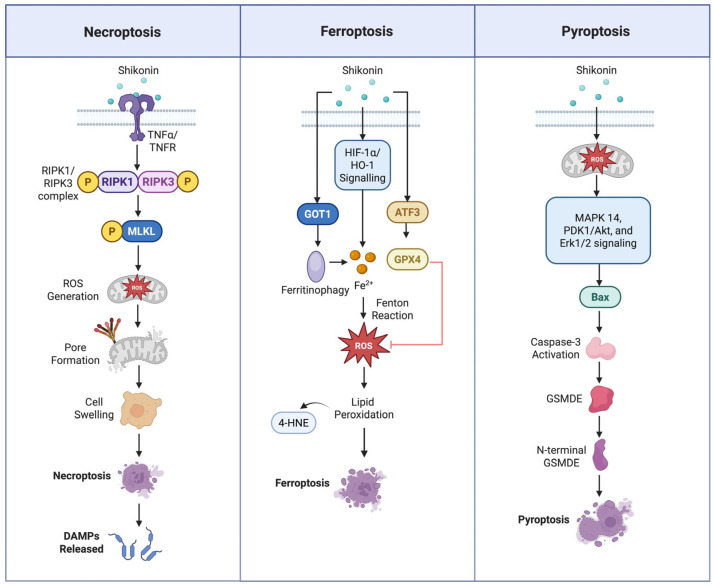
Shikonin-induced regulated cell death through necroptosis, ferroptosis and pyroptosis.

Necroptosis is a regulated form of cell death typically triggered by tumour necrosis factor-α (TNFα) or Toll-like receptors (TLRs) under conditions of caspase-8 inhibition (Figure 3). It is primarily mediated by the phosphorylation of receptor-interacting protein kinase 1 (RIPK1) and receptor-interacting protein kinase 3 (RIPK3), which subsequently phosphorylate mixed lineage kinase domain-like protein (MLKL), resulting in necrosome assembly, plasma membrane rupture, and the release of damage-associated molecular patterns (DAMPs) [121,122,123].

Recent studies have shown that shikonin induces necroptosis in various cancer cell types, including osteosarcoma, glioma, and chronic myeloid leukaemia, through mechanisms involving ROS generation, loss of MMP, and RIPK1/RIPK3 activation [124,125]. In glioma cells (SHG-44, U87, U251), shikonin upregulated RIPK1 and RIPK3 expression via excessive ROS production. By treating mitochondrial superoxide cleaner MnTBAP in these glioma cell lines, the ROS generation decreased, while the combination treatment of RIP1/RIP3 inhibitor and shikonin caused RIP1/RIP3 activation and necrosome assembly decreased, suggesting shikonin induces RIP1/RIP3 activation, which affects the downstream of ROS generation leading to glioma necroptosis [125].

Other than the SHG-44, U87, and U251 glioma cell lines, shikonin also showed necroptotic effects in the A172 and T98G glioma cell lines by increasing RIP1/RIP3 and MLKL levels [125,126]. In chronic myeloid leukaemia (CML) resistant to tyrosine kinase inhibitors, shikonin downregulated miR-92a-1-5p, a negative regulator of MLKL, thereby activating the RIPK1/RIPK3/MLKL signalling axis to induce necroptosis [124].

In Jurkat T-cell leukaemia cells, shikonin triggered a RIP-1-dependent pathway during early apoptosis, suggesting that shikonin induces an RIP1-dependent necroptotic pathway, which will affect the downstream of ROS generation leading to apoptosis [127]. In pancreatic cancer cells (AsPC-1 and PANC-1), shikonin dose-dependently induced both apoptosis and necroptosis. Interestingly, combined treatment with the necroptosis inhibitor Nec-1 and the apoptosis inhibitor zVAD in AsPC-1 cells shifted cell death predominantly toward necroptosis, indicating that shikonin can activate both pathways simultaneously [128].

Another research showed that shikonin induced more predominant necroptotic death in doxorubicin (DX)-resistant prostate cancer cell lines. They found that the combined administration of the Nec-1 inhibitor and shikonin results in reversed anti-growth effects of shikonin in all parental and DX-resistant prostate cancer cell lines [108]. In breast adenocarcinoma MCF-7 cells, shikonin irreversibly inhibited the antioxidant enzyme thioredoxin reductase 1 (TrxR1), inducing oxidative stress and necroptosis, which was prevented by Nec-1 co-treatment. This suggests that targeting TrxR1 may sensitise cancer cells to shikonin-induced necroptosis [129].

Briefly, these findings indicate that shikonin can trigger necroptosis, often in conjunction with apoptosis, across diverse cancer types through pathways involving RIPK1/RIPK3/MLKL activation, ROS generation, and redox system disruption.

### 3.5. Induction of Ferroptosis

Ferroptosis is an iron-dependent form of regulated cell death mechanism that is mediated by iron, lipids, and ROS, which will affect mitochondrial membrane structure and mitochondrial volume [121,122,123]. Shikonin’s antitumor effects extend beyond apoptosis and necroptosis, as it has also been shown to induce ferroptosis in multiple myeloma (MM) through glutamic-oxaloacetic transaminase 1 (GOT-1)-mediated ferritinophagy, resulting in lactate dehydrogenase release and subsequent cell death [130]. In small cell lung cancer (SCLC), shikonin triggered ferroptosis by increasing oxidative stress and suppressing the antioxidant enzyme GPX4 via an ATF3-centred, epigenetically regulated pathway [131]. Moreover, in osteosarcoma MC63 cells, shikonin simultaneously induced apoptosis and ferroptosis, with its ferroptotic effect mediated by mitochondrial ROS (MitoROS)-triggered HIF-1α/HO-1 signalling. This led to iron overload, oxidative stress, and depletion of mitochondrial antioxidant defence enzymes [132]. In short, these findings indicate that shikonin can induce ferroptosis in osteosarcoma and small cell lung cancer, expanding its spectrum of regulated cell death mechanisms beyond apoptosis and necroptosis (Figure 3).

### 3.6. Induction of Pyroptosis

Pyroptosis is a proinflammatory form of regulated cell death pathway that can be triggered by caspase -1, -3, -4, and -5 and is mediated through three cellular pathways, which are granzyme-mediated, canonical, and non-canonical pathways [121,122,123]. Shikonin has demonstrated broad antitumor effects, including the induction of pyroptosis. In human gastric cancer (GC) cell lines SGC-7901 and BGC-823, shikonin induced pyroptosis in a time- and dose-dependent manner via a gasdermin E (GSDME)-mediated pathway. Mechanistically, shikonin increased intracellular ROS, resulting in Bax/caspase-3 activation while concurrently inhibiting MAPK14/p38α-regulated autophagy, thereby amplifying pyroptosis in gastric cancer cells [133].

Additionally, in the EGFR-T790M-mutant drug-resistant NSCLC cell line H1975, shikonin induced both pyroptosis and apoptosis by inhibiting PGE2-induced downstream signalling pathways, including PDK1/Akt and Erk1/2. Co-treatment with shikonin, the necroptosis inhibitor Nec-1, the apoptosis inhibitor zVAD, and the ferroptosis inhibitor Fer-1 revealed compromised cell membranes, confirming that shikonin triggers both pyroptosis and late-stage apoptosis in H1975 cells [93]. In brief, these findings indicate that shikonin is capable of inducing pyroptosis in gastric cancer and non-small cell lung cancer, further expanding its spectrum of regulated cell death mechanisms.

### 3.7. Inhibition of Tumour Metabolism

Tumour cells undergo metabolic reprogramming to meet the increased energy and biosynthetic demands required for rapid proliferation and metastasis [134]. One of the main altered metabolic processes is aggravated aerobic glycolysis, also known as the Warburg effect. It is characterised by increased glucose uptake in cancer cells that preferentially undergo aerobic glycolysis despite sufficient oxygen availability, leading to rapid ATP and lactate production [135]. The resulting acidic TME promotes metastatic cell proliferation and activates proliferation-associated factors such as transforming growth factor β (TGF-β) [136].

PKM2, a key glycolytic enzyme isoform, is frequently upregulated in cancer and plays a central role in metabolic and transcriptional regulation [137]. PKM2 exists in multiple conformations, including inactive monomers, active tetramers, and nuclear dimers. The dimeric form mainly plays a role in modulating cellular proliferation but is also able to stimulate the expression of transcription factors associated with glycolysis [138,139]. It is also able to promote the Warburg effect by favouring glucose-derived carbon utilisation for macromolecular biosynthesis while tetramer PKM2 is highly active and stimulates rapid ATP production via oxidative phosphorylation [140].

Shikonin has been shown to disrupt tumour metabolic reprogramming by directly inhibiting PKM2 activity and expression. Long et al. demonstrated that shikonin-loaded nanoparticles optimised with hyaluronic acid reduced lactate production in CT26 colorectal cancer cells through PKM2 inhibition. Shikonin was able to remodel the tumour immune microenvironment (TIME) by inhibiting lactate-induced immunosuppression and stimulating cancer cell apoptosis [136].

In NSCLC lines A549 and PC9, shikonin at doses of 4.0 µM, 6.0 µM, and 8.0 µM inhibited glucose uptake and lactate production. In the same study, Dai et al. confirmed that shikonin’s antiglycolytic properties were exerted via inhibiting PKM2 by observing its effectiveness in PKM2-deficient NSCLC cells [141]. Another study by Zhou et al. further proved shikonin’s involvement in inhibiting PKM2 activity by attenuating the guanosine triphosphate binding protein 4 (GTPBP4)-PKM2-dependent regulatory axis in glycolytic reprogramming in HCC. Tumours collected from mice xenografted with HCC cell lines showed upregulated levels of GTPM4, an inducer of dimer PKM2 production, which was significantly correlated with poor prognosis, increased metastasis and tumour progression, and increased aerobic glycolysis contributing to the Warburg effect. However, Western blot and immunoprecipitation tests demonstrated that shikonin was able to completely inhibit GTPB4-PKM2-induced glycolytic activity, thus suppressing HCC growth and metastasis [140].

Consistent with previous studies, Zhang et al. found that shikonin inhibited PKM2 activity in a dose-dependent manner in nasopharyngeal carcinoma cells, accompanied by significantly reduced ATP levels, lactate production, and glucose uptake. This demonstrates shikonin’s ability to alter the glycolytic flux and TME by inhibiting PKM2 [116]. Huang et al. observed via size-exclusion chromatography that shikonin inhibited the formation of PKM2 dimer and tetramer isoforms. Shikonin binds to the PKM2 isoforms and modulates PKM2 aggregation into a polymer, thus inhibiting its activity [138].

Additionally, it was also found that shikonin could selectively inhibit the formation of dimer PKM2 forms by binding to the tetramer forms and stabilising them [138,139]. One such factor is hypoxia-inducible factor 1-alpha (HIF-1α), which encodes for the production of glycolytic enzymes, glucose transporters GLUT1, and PKM2 itself [139,141]. The inhibition causes downregulated expression of prolyl hydroxylase 3 (PHD3), a HIF-1⍺ coactivator, thus dysregulating the PHD3/HIF-1⍺ positive feedback loop [86]. Other than via the direct inhibition of PKM2, shikonin also acts on endocan, also known as the endothelial cell specific molecule 1 (ESM1), which facilitates the dimerisation of PKM2 [142].

Shikonin has also been found to inhibit aerobic glycolysis via the inhibition of NHE1, a proton efflux pump, resulting in an acidic tumour cell intracellular microenvironment [117]. While an acidic intercellular microenvironment further aggravates metastasis, an acidic intracellular microenvironment inhibits DNA synthesis initiation, a process requiring an alkaline environment, consequently suppressing cell proliferation [117,136]. Additionally, shikonin exerts inhibitory properties on 6-phosphofruto-2-kinase/fructose-2,6-biphosphate (PFKFB2) expression, an enzymatic regulator of the fructose-2,6-bisphosphate synthesis and degradation in glycolysis [143].

**Figure 4:**
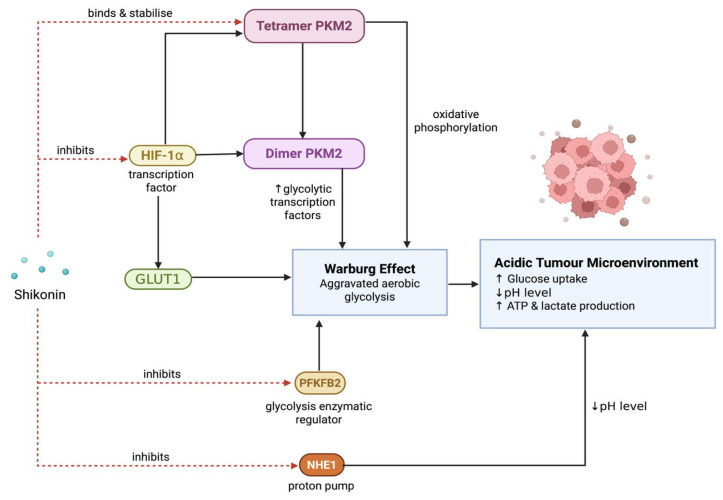
Shikonin suppresses tumour metabolism by inhibiting PKM2 and glycolysis. Key in-creases (↑) and decreases (↓) in molecular markers are indicated.

Overall, shikonin is able to suppress tumour metabolism by acting on various mechanisms inhibiting the Warburg effect. It mainly acts on the glycolytic enzyme PKM2 via different approaches: direct inhibition via aggregation, preventing the formation of its tetramer and dimer isoforms, and targeting its transcriptional factor, HIF-⍺ (Figure 4). Additionally, shikonin interferes with other factors of the glycolytic cycle as well, such as the inhibition of NHE1 and PFKFB2. This inhibition allows shikonin to alter the tumour’s intracellular and intercellular microenvironment, specifically by altering the pH levels against favouring tumour growth. Shikonin’s mechanism in suppressing tumour metabolism is well established and has consistently shown improved prognosis over multiple studies, with lowered production of ATP and lactate and glucose uptake.

## 4. Combination Therapy

### 4.1. Shikonin as a Chemosensitiser: Enhancing Chemotherapy and Overcoming Resistance

The therapeutic limitations of single-agent chemotherapy have driven widespread interest in combination strategies that can simultaneously target multiple cancer pathways [144]. Shikonin functions as a potent chemosensitiser, enhancing the efficacy of chemotherapeutic agents while overcoming drug resistance.

#### 4.1.1. Inhibition of PKM2 Expression in Non-Small Cell Lung Cancer

In NSCLC, Dai et al. reported that shikonin significantly enhanced cisplatin sensitivity through dual inhibition of PKM2-mediated glycolysis and exosome signalling [141]. PKM2 is a central regulator of the Warburg effect, sustaining tumour growth through aerobic glycolysis and biosynthesis, while also promoting exosome release via non-metabolic activation of SNAP-23. These exosomes carry oncogenic cargo that remodels the TME, driving angiogenesis, metastasis, and immune evasion. Targeting PKM2 could therefore disrupt both metabolic reprogramming and exosome-mediated signalling, providing a dual approach to suppress tumour progression [145,146]. Shikonin suppresses PKM2 expression by inhibiting HIF-1α transcription and the splicing factors that drive PKM2 isoform selection. This disruption of the PKM2/HIF-1α axis reduces inflammation and oxidative stress [138]. Notably, shikonin exerted potent anti-tumour effects in NSCLC cells, with IC_50_ values at 24 h of 5.739 µM in A549 and 6.302 µM in PC9 cells. At 8 µM, shikonin reduced PK activity by ~33% within 20 min. In A549 cells, it markedly suppressed glucose uptake (~67%) and lactate production (~57%), compared with more modest reductions in PC9 cells (~24% and ~40%), reflecting greater sensitivity in A549. This metabolic inhibition corresponded with decreased PKM2 expression and activity, without changes in MCT1 or LDHA. PKM2 knockdown abolished these anti-glycolytic and anti-proliferative effects, confirming the dependence of shikonin’s action on PKM2 inactivation [141].

Shikonin also impaired exosome release, reducing exosomal PKM2 secretion by ~69%, while exosome blockade with GW4869 produced similar restoration of cisplatin sensitivity. This decrease in exosome signalling coincided with a strong pro-apoptotic effect, with ~650% and ~525% increases in apoptosis at 6 µM observed in A549 and PC9 cells, respectively. Importantly, cisplatin-resistant NSCLC cells transferred resistance to cisplatin-sensitive A549 cells through exosomal PKM2, as shown by a ~72% reduction in cisplatin efficacy when A549 cells were exposed to cisplatin alone. Shikonin effectively reversed this acquired resistance in a dose-dependent manner, restoring up to ~100% of cisplatin sensitivity in cells cultured in conditioned media, confirming its ability to block PKM2-mediated resistance transfer. In nude mice inoculated with A549 cells, intraperitoneal co-treatment with shikonin and cisplatin markedly enhanced cisplatin sensitivity through metabolic reprogramming, resulting in the greatest reductions in tumour burden and ^18^F-FDG uptake compared with monotherapies. Shikonin downregulated PKM2 and Glut1 expression, as confirmed by immunohistochemistry (IHC), corresponding with a >50% decrease in glucose uptake on ^18^F-FDG PET/CT (SUVmax), and suppressed exosomal PKM2 release. Notably, inhibition of exosome secretion with GW4869 or direct targeting of exosomal PKM2 restored cisplatin responsiveness, underscoring shikonin’s dual action on intracellular metabolism and intercellular resistance transmission. These combined effects led to >70% reductions in tumour volume and weight [141].

#### 4.1.2. Upregulation of HMOX1-Induced Ferroptosis in Ovarian Cancer

Ni et al. demonstrated that shikonin synergises with cisplatin to overcome resistance in ovarian cancer by enhancing ferroptosis, an iron-dependent form of cell death driven by lipid peroxidation [147]. This approach is particularly effective against aggressive, apoptosis-resistant tumour cells, exploiting their dependence on iron and dysregulated lipid metabolism to induce lethal oxidative damage [148]. The study employed three cisplatin-resistant ovarian cancer cell lines, A2780/DDP, SKOV3/DDP, and OVCAR4/DDP, with average IC_50_ values of 23.46 µM, 50.06 µM, and 18.06 µM, respectively. Compared with cisplatin alone, shikonin in combination therapy significantly upregulated heme oxygenase 1 (HMOX1), as shown by volcano plot analysis, where 23 proteins were downregulated and 35 were upregulated [147]. Increased HMOX1 expression promoted heme breakdown [149] and increased intracellular ferrous iron (Fe^2+^) levels by 50% in A2780/DDP and 100% in SKOV3/DDP cells [150]. Higher Fe^2+^ facilitated ROS production through Fenton reactions, generating hydroxyl radicals that attacked polyunsaturated fatty acids in cell membranes [151]. This triggered ~34% and ~50% increases in ROS levels in A2780/DDP and SKOV3/DDP cells, respectively, leading to ~33% and ~90% increases in lipid peroxidation [147]. The resulting oxidative stress overwhelmed antioxidant defences, compromised membrane integrity, and induced ferroptosis [152,153], thereby restoring cisplatin sensitivity in resistant ovarian cancer cells.

In vivo, the therapeutic synergy was confirmed in a cisplatin-resistant ovarian cancer xenograft model using BALB/c nude mice inoculated with A2780/DDP cells. Shikonin at 0.8 mg/kg combined with cisplatin at 3.0 mg/kg suppressed tumour growth by over 67% compared to monotherapies, without causing weight loss or hepatorenal toxicity. IHC confirmed 3-fold upregulation of ferroptosis markers, including HMOX1, TFRC, and POR. This dual-action mechanism, inducing iron-dependent cell death while maintaining metabolic safety, highlights shikonin as an effective adjunct to platinum-based chemotherapy in overcoming resistance in ovarian cancer [147].

#### 4.1.3. Upregulation of p53-Mediated Apoptosis in Oesophageal Cancer

Besides cisplatin, shikonin also demonstrates synergistic effects with other chemotherapeutic drugs, such as paclitaxel. Du et al. investigated shikonin’s ability to sensitise oesophageal squamous cell carcinoma (ESCC) to paclitaxel using KYSE270 and KYSE150 cell lines. In vitro, co-treatment with shikonin and paclitaxel increased p53 expression by approximately 200% [154]. P53 is a tumour suppressor that regulates genes involved in cell cycle arrest, apoptosis, senescence, autophagy, and DNA repair, and its upregulation led to markedly higher apoptosis, with a ~367% increase compared to either agent alone [154,155]. This was supported by enhanced caspase-3 cleavage, detected by Western blotting, and by nuclear fragmentation, both of which are established hallmarks of apoptosis. Caspase-3 acts as a key executioner protease, mediating programmed cell death by cleaving critical structural and regulatory proteins, while nuclear fragmentation reflects chromatin breakdown during late-stage apoptosis [156,157,158]. Moreover, p53 upregulation suppressed BCL2 expression by about 38% [154]. BCL2 is an anti-apoptotic protein that confers resistance to chemotherapy, and its reduction enhances sensitivity to treatment [159,160]. Whereas either shikonin or paclitaxel alone had minimal effects on p53 and BCL2, their combination significantly increased p53 and decreased BCL2, underscoring their cooperative role in promoting apoptosis [154].

#### 4.1.4. Inhibition of PAK1 Expression in Pancreatic Cancer

Pancreatic cancer has a poor prognosis, with gemcitabine-based therapy providing only limited survival benefits. Ji et al. investigated whether combining shikonin with gemcitabine could enhance therapeutic efficacy by targeting p21-activated kinase 1 (PAK1) [161], an oncogenic kinase frequently overexpressed in pancreatic cancer that drives proliferation, survival, invasion, and therapy resistance [162,163]. Using the PANC-1 and BxPC-3 cell lines, shikonin was identified as a novel PAK1 inhibitor, with IC_50_ values of 1.800 ± 0.013 μM in PANC-1 and 3.183 ± 0.321 μM in BxPC-3 cells. Western blot analysis confirmed that shikonin inhibited PAK1 phosphorylation and suppressed its downstream signalling pathways, including mTOR, MEK1, and c-RAF. Apoptosis was induced dose-dependently, with 3, 5, and 10 μM treatments causing 14.66%, 83.35%, and 90.50% apoptosis in BxPC-3 cells and approximately 7%, 14%, and 17% in PANC-1 cells, respectively. Notably, shikonin markedly sensitised BxPC-3 cells to gemcitabine, reducing the IC_50_ values from 14.22 to 2.18 μM, with strong synergism demonstrated by combination index values of 0.18. These findings highlight shikonin as a potent bioactive inhibitor of PAK1 and a promising candidate for combination therapy in pancreatic cancer [161].

Taken together, these studies underscore shikonin’s broad capacity to enhance chemotherapeutic efficacy by targeting diverse resistance mechanisms across multiple malignancies. Mechanistically, it disrupts tumour survival through dual blockade of PKM2-mediated glycolysis and exosome signalling in NSCLC, ferroptosis induction in cisplatin-resistant ovarian cancer, p53-dependent apoptosis in ESCC, and PAK1 inhibition in pancreatic cancer. Clinically, shikonin consistently restores drug sensitivity and amplifies cytotoxicity, highlighting its potential as a versatile adjunct to combination chemotherapy. These findings warrant further preclinical and clinical evaluation to establish its translational value in overcoming therapy resistance and improving patient outcomes.

### 4.2. Endocrine-Targeted Therapy and Resistance Modulation by Shikonin

Targeted therapies have transformed breast cancer treatment, yet their efficacy is often limited by the emergence of resistance mechanisms that diminish clinical benefit. Resistance can develop through on-target genetic alterations that impair drug binding, such as EGFR T790M and ALK mutations, or through activation of alternative survival pathways, including MET amplification and KRAS mutations, which circumvent the inhibited signalling axis [164,165].

#### 4.2.1. Activation of Mitochondrial-Mediated Apoptosis in Breast Cancer

Shikonin has been shown to enhance the efficacy of endocrine-targeted therapy in both oestrogen receptor–positive (ER^+^) and –negative (ER^−^) breast cancers by modulating apoptotic and survival pathways. Lin et al. reported that combining shikonin with 4-hydroxytamoxifen (4-OHT) in MCF-7 (ER^+^) and MDA-MB-435S (ER^−^) cells produced maximum inhibition rates of 47% and 66%, respectively, surpassing the effects of either drug alone [166]. Mechanistically, shikonin targeted mitochondrial complex II (succinate dehydrogenase), causing electron transport chain dysfunction and excessive superoxide production, which increased ROS levels 2.3-fold in MCF-7 and 1.8-fold in MDA-MB-435S [166,167]. The resulting oxidative stress damaged mitochondrial components and triggered mitochondrial permeability transition pore opening, leading to loss of MMP by ~77% in MCF-7 and ~60% in MDA-MB-435S [166,168]. Dissipation of MMP initiated intrinsic apoptosis through cytochrome c release and Apaf-1/caspase-9 apoptosome formation, with concurrent Smac/DIABLO release neutralising IAPs and activating the caspase cascade [169].

Combination treatment increased early apoptosis by 22.9% in MCF-7 and late apoptosis by 26.3% in MDA-MB-435S. Additionally, shikonin suppressed PI3K expression by ~33% in MCF-7 and ~20% in MDA-MB-435S, while reducing AKT activity by ~68% only in MCF-7, implicating the PI3K/AKT/caspase-9 axis as a key mechanism in ER^+^ cells. In vivo, in a BALB/c mouse model bearing MCF-7 xenografts, the combination of shikonin (1.5 mg/kg) and 4-OHT (3 mg/kg) produced superior antitumour efficacy, achieving 76.65% tumour growth inhibition versus 57.20% for shikonin and 45.44% for 4-OHT alone. Tumour weight was reduced by 58% and 47% compared to 4-OHT and shikonin monotherapies, respectively. While shikonin alone caused mild adverse effects, the combination treatment maintained a favourable safety profile, with no significant body weight loss or renal toxicity. Histopathological analysis showed enhanced apoptosis and reduced malignancy in the combination group, as confirmed by H&E staining and IHC. These findings highlight the synergistic potential of shikonin to overcome endocrine resistance in ER^+^ breast cancer, with a 1:2 dose ratio (shikonin:4-OHT) emerging as an effective and tolerable regimen [166].

#### 4.2.2. Restoration of the uc.57–BCL11A Axis Overcomes Tamoxifen Resistance

Complementing these findings, Zhang et al. investigated shikonin as a potential modulator of tamoxifen (TAM) resistance by targeting resistance-associated pathways and ultra-conserved long non-coding RNAs (lncRNAs) [170]. LncRNAs are non-protein-coding transcripts longer than 200 nucleotides that regulate gene expression and contribute to cancer progression by modulating chromatin structure, transcriptional programmes, and signalling pathways. Certain lncRNAs also drive tumour metastasis and therapy resistance through interactions with epigenetic complexes and hormone receptors [171]. Using TAM-resistant MCF-7R and parental MCF-7 cell lines, the study showed that MCF-7R cells expressed ~80% less of the ultra-conserved lncRNA uc.57 and ~280% more of its target gene BCL11A compared with TAM-sensitive cells, linking uc.57 downregulation and BCL11A upregulation to TAM resistance. Shikonin treatment restored uc.57 expression, which suppressed BCL11A and consequently inhibited the PI3K/AKT and MAPK pathways by ~50% [170].

The MAPK pathway promotes growth and angiogenesis through RAS/RAF/ERK signalling, while PI3K/AKT enhances survival and epithelial–mesenchymal transition through lipid-mediated signals. Frequent activation and crosstalk of these pathways contribute to tumour aggressiveness and endocrine therapy resistance, making their dual inhibition an important therapeutic goal [172,173,174]. Functionally, shikonin plus TAM suppressed MCF-7R cell growth by 69% in vitro, whereas TAM alone had no effect. In human breast tissue-derived SCID mouse models of TAM-resistant ER-positive breast cancer (MCF-7R xenografts), shikonin at 1.5 mg/kg combined with TAM (3 mg/kg) reduced tumour volume by over 50% compared to shikonin alone, effectively overcoming TAM resistance. Parallel experiments with uc.57-overexpressing MCF-7R-lv-uc.57 xenografts confirmed this mechanism, as TAM monotherapy significantly inhibited tumour growth in uc.57-positive tumours, recapitulating the effect of the shikonin–TAM combination. The five-week treatment caused no observable toxicity, with stable body weight and normal ALT/AST levels [170].

Together, these findings demonstrate shikonin’s ability to enhance endocrine-targeted therapies by disrupting both mitochondrial metabolism and transcriptional resistance pathways. Mechanistically, it induces mitochondrial dysfunction, elevates ROS production, and suppresses PI3K/AKT signalling in ER^+^ breast cancer, while restoring the uc.57–BCL11A axis and dual blockade of PI3K/AKT and MAPK pathways in tamoxifen-resistant models. Clinically, shikonin potentiates 4-OHT efficacy, restores tamoxifen responsiveness, and reduces tumour burden, supporting its role as a promising adjuvant strategy. These results highlight the translational value of shikonin in overcoming endocrine resistance, with uc.57 and BCL11A emerging as candidate biomarkers for future therapeutic development.

### 4.3. Shikonin Enhances Immunotherapy via Immunogenic Cell Death

Immunogenic cell death (ICD) is a regulated form of tumour cell death that stimulates adaptive immunity through the release of DAMPs such as calreticulin (CRT), ATP, HMGB1, and heat shock proteins. These signals recruit and mature dendritic cells (DC), enhance tumour antigen presentation, and activate cytotoxic T lymphocytes (CTL), establishing long-term immune memory. Shikonin induces ICD via ROS-driven apoptosis and necroptosis, marked by CRT exposure, ATP release, and HMGB1 secretion. Importantly, tumour lysates from shikonin-treated cells, especially when combined with pathogen-associated molecular patterns (PAMPs), further promote dendritic cell maturation and stimulate Th1 and Th17 responses, thereby reinforcing anti-tumour immunity [175,176].

In TNBC, Shahsaari et al. reported that shikonin enhances anti–PD-1 immune checkpoint therapy by inducing RIPK1/RIPK3-dependent necroptosis [177]. Necroptosis is a caspase-independent programmed cell death triggered when death receptors such as TNFR1 or cellular stress activate RIPK1, which phosphorylates RIPK3 to form the necrosome complex. This drives MLKL oligomerisation, membrane permeabilisation, and inflammatory necrosis [178,179,180]. Shikonin showed an IC_50_ of 3.586 μM at 12 h and acted by generating ROS, disrupting MMP (~83%), and upregulating RIPK1 (~35%) and RIPK3 (~40%). It demonstrated plasticity between death pathways, with necroptosis dominating when apoptosis was blocked by Z-VAD-FMK (necrosis ~20%, apoptosis ~2%) and apoptosis prevailing when necroptosis was inhibited by Nec-1 (necrosis ~3%, apoptosis ~39%). Caspase-3 and -8 activity increased by ~633% and ~288%, respectively, confirming strong apoptotic induction. Notably, shikonin’s capacity to trigger immunogenic necroptosis enhanced the efficacy of PD-1 blockade by reshaping the TME to favour immune infiltration and recognition. By targeting mitochondrial dysfunction and activating RIPK1/RIPK3/MLKL while preserving caspase-dependent apoptosis, shikonin presents a dual cytotoxic and immunomodulatory strategy for treating aggressive breast cancers [177].

Supporting evidence from Chen et al. demonstrated that shikonin synergises with PD-1 blockade to overcome immunotherapy resistance via a PKM2-ROS-Hsp70 immunogenic axis [181]. Hsp70 acts as a dual-function chaperone, maintaining proteostasis and inhibiting apoptosis intracellularly while serving extracellularly as a DAMP to activate antigen-presenting cells (APCs), enhance NK cell cytotoxicity, and stimulate pro-inflammatory cytokine production. Its context-dependent functions, both promoting tumour growth and facilitating immune recognition, make Hsp70 a compelling therapeutic target for inhibition to disrupt cancer survival or exploitation to boost ICD and anti-tumour immunity [182,183,184]. Shikonin induced dual cell death in CT26 cells, combining dose-dependent apoptosis (12.47% at 5 μM and 20.17% at 10 μM) with PKM2-dependent ROS-mediated ICD, confirmed by reversal with PKM2 silencing and NAC inhibition. It specifically enhanced CRT surface exposure (22.13 ± 0.153% at 5 μM; 23.70 ± 0.265% at 10 μM) and Hsp70 expression (32.61 ± 11.260) without significant changes in total CRT or HMGB1 levels or soluble HMGB1 release, as shown by Western blot and ELISA. These changes remodelled the tumour microenvironment, increasing CD8^+^ T-cell infiltration (2.52 ± 0.659%) and dendritic cell recruitment (4.02 ± 2.112%). In vivo, CT26 cells were subcutaneously inoculated into the right inguinal region of SPF female BALB/c mice, and treatment was initiated once tumours reached approximately 62.5 mm^3^. Shikonin was administered intraperitoneally at 3 mg/kg, administered every 2 days for a total of seven doses, while anti-PD-1 antibody was given intraperitoneally at 50 µg per mouse on days 5 and 9. This combination significantly enhanced anti-PD-1 efficacy, reducing tumour volume by 40% compared to PD-1 monotherapy (294.1 ± 231.2 mm^3^ vs. 489.2 mm^3^) and by 72% versus untreated controls (1048 ± 1016 mm^3^). The more than 3.5-fold reduction underscores shikonin’s therapeutic potential as an adjuvant to checkpoint inhibitors for microsatellite-stable colorectal cancers where PD-1 monotherapy typically fails [181].

Shikonin represents a promising phytochemical for immunotherapy-resistant clear cell renal cell carcinoma (ccRCC), demonstrating a distinctive triple mechanism of action when combined with ipilimumab. In the SKRC17 and RCC53 cell lines, the latter derived from a patient with stage IV disease (pT2N1MxG2 to G3), Lyu et al. reported that shikonin directly suppressed cancer stem cells (CSCs), reducing migration and invasion by 50% and increasing apoptosis by approximately 344%, with an IC_50_ of 1.32 μM. It also reprogrammed the immune microenvironment by decreasing FoxP3^+^ regulatory T cells (Tregs) by 70% and enhancing CD4^+^ and CD8^+^ T cell activation 2.5-fold in patient peripheral blood mononuclear cell (PBMC) co-cultures. In addition, it modulates CSC-immune biomarkers, including VCAM1, IL8, and CXCL1. Integrated NanoString profiling and network pharmacology identified 19 shared molecular targets underpinning the shikonin–ipilimumab synergy, with VCAM1 emerging as a predictive biomarker. High VCAM1 expression correlated with a 3-fold improvement in survival and increased NK/T-cell infiltration in TCGA data, although its prognostic significance was reversed under CTLA-4 blockade. Unlike current VEGF-TKI/ICI regimens limited to intermediate-poor risk IMDC patients, this combination uniquely addresses CSC-driven resistance through PKM2-mediated metabolic rewiring and ROS-dependent CSC elimination while enhancing tumour immunogenicity. Clinical translation is further supported by CSC marker downregulation (IL8, CXCL1, VCAM1), activation of immune and NK-cell signalling pathways, and stratification potential via Immunophenoscore, positioning the combination for future trials in ICI-refractory ccRCC [185].

In aggregate, these studies reveal that shikonin can enhance immunotherapy by reshaping the TIME. Mechanistically, it reduces lactate-induced immunosuppression, promotes DC activation, and stimulates cytotoxic T-cell responses while concurrently inhibiting immune checkpoints such as PD-1/PD-L1. In addition, shikonin has been reported to induce ICD, characterised by calreticulin exposure, ATP release, and HMGB1 secretion, thereby further enhancing antigen presentation and adaptive immune activation. Clinically, shikonin has shown the capacity to restore immune sensitivity and strengthen the efficacy of checkpoint inhibitors, highlighting its value in overcoming immune evasion. These findings warrant further preclinical validation and clinical trials to define optimal combination strategies and evaluate their safety and translational impact in immuno-oncology.

### 4.4. Shikonin Derivative β, β-Dimethylacrylshikonin Enhances Radiotherapy

To investigate the radio-sensitising potential of shikonin, Kim et al. examined its analogue β, β-dimethylacrylshikonin and found that it markedly enhanced cancer cell sensitivity to ionising radiation (IR) through ROS-mediated mechanisms. In vitro studies across several cancer cell lines showed that HCT116 colorectal carcinoma cells exhibited the greatest reduction in viability (~44%) when pretreated with the analogue before IR, while LN428 glioma (~13%), H460 NSCLC (~26%), and A549 lung carcinoma (~18%) cells displayed only minor effects. Although the compound alone induced limited apoptosis, its combination with 5 Gy IR synergistically increased apoptotic cell death (~200%). This effect was linked to an approximately 123% increase in ROS and a ~196% increase in DNA damage, with Western blot and immunofluorescence confirming elevated γ-H2AX expression. The ROS scavenger N-acetylcysteine suppressed both ROS accumulation and apoptosis (~27%), with IC_50_ confirming ROS dependence. In HCT116 xenograft models, ββ dimethylacrylshikonin showed remarkable radiosensitisation, with combination therapy using 5 Gy ionising radiation and the analogue achieving near complete tumour growth arrest (<500 mm^3^), compared to ~50% suppression with radiation alone and 3000 mm^3^ in untreated controls. This 6-fold enhancement of radiation efficacy occurred without observable toxicity, indicating a favourable therapeutic window for colorectal cancer and potential for significant radiation dose reduction in clinical settings. The analogue’s superiority over native shikonin underscores the value of structure activity optimisation for radiotherapy adjuvants [150].

Together, the evidence underscores β, β-dimethylacrylshikonin as a potent radiosensitiser that augments the therapeutic efficacy of ionising radiation through ROS-mediated DNA double-strand breaks, repair pathway disruption, and apoptosis induction. Its pronounced activity in HCT116 colorectal carcinoma, particularly in p53 mutant models, highlights its potential to overcome radioresistance, while tumour type specificity observed in glioma and lung carcinoma lines suggests the need for biomarker-driven patient selection. Importantly, in vivo studies confirm its strong efficacy and favourable safety profile, reducing tumour burden without exacerbating normal tissue toxicity. These findings support further exploration of β, β-dimethylacrylshikonin and related shikonin analogues as promising adjuvant strategies to enhance radiotherapy outcomes. Table 3 summarises the synergistic outcomes of shikonin in combination with various anticancer ther-apies across multiple cancer types.

## 5. Preclinical and Clinical Studies

Several in vivo preclinical studies have been conducted to evaluate the potential of shikonin as an anticancer agent based on its well-characterised mechanisms. Shikonin has demonstrated consistent anticancer effects across diverse in vivo tumour models, supporting its potential for clinical application. Moreover, shikonin shows promise in enhancing the efficacy of existing anticancer therapies, particularly in overcoming drug resistance when used in combination strategies. Table 4 summarises the preclinical and clinical studies evaluating shikonin and its derivatives in various cancer models.

Bhat et al. reported that shikonin significantly inhibited tumour growth in xenografted mice bearing human melanoma A375 cells, with Western blot analysis confirming increased expression of apoptotic proteins. At a dose of 5.0 mg/kg, shikonin-treated mice exhibited greater tumour suppression than those treated with dacarbazine, a standard chemotherapeutic agent [111]. Similarly, Zhu et al. demonstrated that combined treatment with shikonin and quercetin in a B16F10 melanoma mouse model significantly reduced the number of lung metastatic nodules without adverse effects, partially attributed to the inhibition of platelet–tumour interactions and glycolysis via PKM2 suppression [186].

Orthotopic mouse models of breast cancer treated with shikonin were found to have significant tumour growth inhibition and reduced tumour cell invasion in a study conducted by Pander et al. TNBC has also been found to be responsive to treatment with shikonin via mechanisms such as the inhibition of PDK1 [17]. TNBC cells were particularly sensitive to shikonin, which exerted antitumor effects via PDK1 inhibition and EMT suppression, two pathways closely associated with drug resistance and aggressive metastasis [187]. Li et al. developed an antigen-targeted nanoparticle system for co-delivery of shikonin, TGF-β, and small interfering RNA. Flow cytometry and confocal microscopy revealed that 21.8% of treated cells underwent ICD, as indicated by calreticulin exposure. This nanoparticle system demonstrated optimal biodistribution, prolonged circulation time, and specific tumour cell targeting in vivo [188].

Zhang et al. conducted an in vivo study using BALB/c mice xenografted with head and neck squamous carcinoma cells targeting the FAM83A/PKM2 axis by suppressing PKM2 expression with shikonin. The study elucidated a newfound relationship between the involvement of FAM83A in PKM2 regulation and tumour progression and indicated that shikonin could be a promising therapeutic treatment targeting the FAM83A/PKM2 axis in treating head and neck squamous cell carcinomas (HNSCC) [189].

There have also been animal studies based on cervical and ovarian cancer treatment with shikonin, which showed shikonin’s capabilities to suppress tumour progression by inhibiting metastasis and stimulating apoptosis [17]. Shikonin has also been found to improve treatment for cisplastin-resistant ovarian cancer cells by enhancing its cytotoxicity [147]. There have also been investigations into shikonin’s effectiveness in oral cancer, where Min et al. showed that oral cancer growth was successfully inhibited in a mouse model [190]. Shikonin derivatives have also been investigated, such as β-hydroxyisovaleryl-shikonin by Zeng et al., who observed increased cell apoptosis and ROS production in pancreatic cancer cells [49]. Due to its glycolysis-inhibiting property, it induces weight loss in animal models and causes skin sensitisation [55]. Shikonin is also known to exert hepatotoxic effects due to its inhibitory effects against uridine 5’-diphosphate-glucuronosyltransferase, making it unsuitable for clinical usage despite its benefits as an anticancer therapeutic agent [36,55]. However, in vivo animal toxicity studies showed that the method of administration helped reduce the shikonin’s toxicity greatly. Oral administration resulted in little to no toxicity, with a >1.0 g/kg LD_50_, while intraperitoneal and intravenous injections yielded results indicating specific toxicity. These findings suggest that tissue-specific targeting and optimised administration routes are critical to mitigating toxicity, although the roles of metabolism and toxicokinetics require further clarification [36].

Another limiting factor is the vague bioavailability of shikonin, as it is easily influenced by patient-dependent factors such as metabolism and toxicokinetics. Establishing preclinical data has shown that shikonin may potentially damage normal tissues if not adequately targeted to cancer cells [55]. Prolonged shikonin treatment has been associated with nuclear membrane damage, although Tabari et al. demonstrated that co-treatment with metformin activated protective pathways such as AMP-activated protein kinase (AMPK), preventing this effect [118,192]. A popular method of targeted delivery of shikonin is with the use of liposomes, such as by Zhu et al. in their shikonin-quercetin study, which also showed adverse effects due to its tendency to accumulate in the liver, resulting in hepatotoxicity rather than the intended targets [186]. A more optimal delivery approach may be via targeted nanoparticles, such as the Xu et al. shikonin-loaded mesoporous polydopamine-based nanoparticles in an emulsion, which allowed improved drug solubility and release rate [193]. Alternatively, Chen et al. utilised hyaluronic acid-modified Fe-MOF nanoparticles as a glycolysis-mediated agent to deliver shikonin to the tumour site and reported improved shikonin solubility alongside combating the toxic nature of shikonin towards other healthy cells exposed [194].

Despite having well-established data in preclinical in vivo trials, there are very few existing and ongoing clinical trials involving the treatment of cancer using shikonin [55]. To date, the only documented clinical trial of the implementation of shikonin as an anticancer therapeutic agent was conducted by Guo et al. in 1991, in which shikonin was prescribed to advanced-stage lung cancer patients [191]. The study reported a tumour reduction of over 25% in diameter and an average survival of 10 months but did not provide detailed information on dosage or adverse effects [191]. Much of these data are outdated and lack detailed information on dosing and administration. This lack of recent and comprehensive clinical data underscores the urgent need for modern clinical trials incorporating advanced drug delivery strategies and informed by preclinical findings to fully evaluate shikonin’s therapeutic potential.

Overall, preclinical studies consistently demonstrate that shikonin and its derivatives exert potent anticancer effects across diverse tumour models, acting through apoptosis induction, glycolysis inhibition, ICD, and enhancement of combination therapies. While these findings highlight its translational promise, concerns regarding hepatotoxicity, limited bioavailability, and patient-dependent pharmacokinetics remain critical barriers. Only one outdated clinical trial has been reported, underscoring the urgent need for well-designed modern clinical studies. Future efforts should prioritise targeted delivery systems, toxicity mitigation, and biomarker-driven patient selection to establish shikonin as a viable adjuvant or stand-alone anticancer therapy.

## 6. Challenges and Future Perspectives

Despite compelling preclinical evidence supporting shikonin’s anticancer potential across multiple tumour types and its promise as a combination therapy candidate, significant barriers continue to hinder its clinical translation. Pharmacokinetic limitations remain a primary challenge, as poor aqueous solubility, rapid metabolism, and variable first-pass effects lead to extremely low systemic bioavailability. These issues are compounded by dose-dependent hepatotoxicity, skin sensitisation, and non-specific accumulation in healthy tissues such as the liver, raising important safety concerns. Such risks underline the need for comprehensive toxicological assessments before advancing to large-scale human trials. Furthermore, while preliminary studies suggest synergistic potential in treatment combinations, supporting evidence remains limited, with few clinical investigations to date. This underscores a persistent gap between promising preclinical findings and real-world application.

Addressing these limitations will require coordinated strategies, including optimised formulations, a clearer understanding of shikonin’s mechanisms, and well-designed clinical trials. One promising direction is tumour-targeted delivery systems, particularly nanoparticles, which can improve aqueous solubility, create prodrug analogues, and enhance selective accumulation at tumour sites. Advances in plant-derived nanomedicine also make it possible to co-encapsulate shikonin with complementary phytochemicals, maximising therapeutic efficacy while limiting off-target toxicity. A strong example is the MUC1@ACS nanocomplex, which combines shikonin with chitosan-coated silver nanoparticles and an MUC1 aptamer for highly specific delivery to TNBC cells. This system achieved a 6.02-fold increase in tumour accumulation compared to free drugs, induced necroptotic ICD via the RIPK3/p-RIPK3/MLKL pathway and triggered potent systemic anti-tumour immunity. As a result, it produced a 68.5% reduction in primary tumour growth and a 91% decrease in metastatic lung and liver nodules, with complete distal tumour regression in some cases. Notably, it showed no significant organ toxicity, highlighting nanoparticles as a viable way to overcome shikonin’s bioavailability and safety barriers [195].

In the longer term, shikonin’s multifaceted mechanisms of action position it as a strong candidate for integration into precision oncology frameworks. Continued progress will depend on sustained multidisciplinary collaboration involving pharmacology and clinical oncology. With strategic optimisation and well-designed clinical trials, shikonin or its derivatives could become valuable therapeutic options for cancers that currently lack effective treatments, potentially transforming patient outcomes. Overall, bridging preclinical promise with clinical validation remains the critical step for establishing shikonin as a translational anticancer therapy.

## 7. Conclusions

In conclusion, although conventional cancer therapies have advanced, persistent challenges such as drug resistance and systemic toxicity continue to limit patient outcomes. Shikonin, a multifunctional dietary phytochemical, exerts potent anticancer activity through multiple forms of regulated cell death, suppression of EMT, and PKM2-driven metabolic disruption. Its ability to synergise with conventional therapeutic strategies presents a promising avenue to overcome therapeutic resistance. However, its clinical application is still restricted by poor bioavailability, dose-dependent hepatotoxicity, and the lack of well-designed clinical trials. Overcoming these obstacles through advanced drug delivery systems, thorough preclinical evaluation, and translational studies will be essential for establishing clinical feasibility. By bridging the principles of traditional medicine with modern oncology, shikonin and its derivatives stand out as compelling candidates for innovative, synergistic cancer therapies, particularly in treatment-resistant malignancies. This review highlights shikonin as a bridge between natural product pharmacology and precision oncology, underscoring its potential to transform future cancer therapy.

## Figures and Tables

**Table 1:** Shikonin derivatives and associated cancer targets.

Shikonin Derivatives	Chemical Structure	Structural Modifications	Cancer Types	Ref.
5, 8-*O*-dimethyl acylshikonin	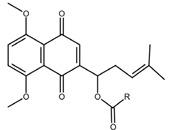	Methylation of phenolic hydroxyl group	Colon cancer Leukaemia Breast cancer	[39]
Shikonin oxime derivative	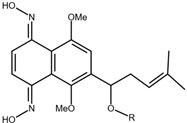	Conversion of carbonyl to oxime group	Breast cancer Leukaemia Prostate cancer	[40]
α-methylbutyrylshikonin	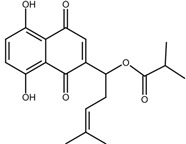	Esterification of hydroxyl group with α-methylbutyryl group	Melanoma Leukaemia	[41,42]
β,-β-dimethylacrylshikonin	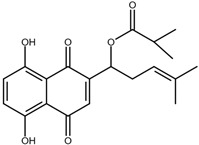	Side chain esterified with dimethylacrylic group	Colorectal cancer Gastric cancer Medullary thyroid cancer	[43,44,45]
Acetylshikonin	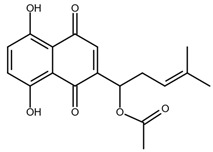	Acetylation of hydroxyl group	Oral squamous cell carcinoma (OSCC) Non-small cell lung cancer (NSCLC) Colorectal cancer	[46,47,48,49]
β-hydroxyisovalerylshikonin	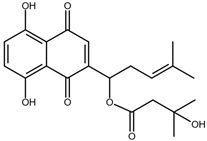	Esterification with β-hydroxyisovaleryl group	Pancreatic cancer Cervical cancer	[49,50]
Deoxyshikonin	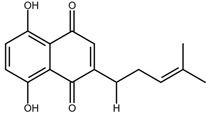	Removal of hydroxyl group	Cervical cancer	[51]
Isobutyrylshikonin	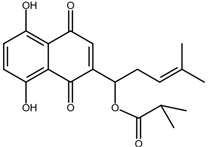	Esterification with isobutyrl group	Oral cancer	[52]

**Table 2:** Shikonin derivatives induced apoptosis through multiple pathways in various cancers.

Shikonin Derivatives	Target Cancer Types	Pathways	Results	Ref.
Acetylshikonin	Leukaemia (K562)	Intrinsic	↑ Cleaved caspase-3; ↑ PARP; ↑ Caspase-9	[101]
β-hydroxyisovaleryl-shikonin	Ovarian (HeLa)	PI3K/AKT/mTOR	↓ PI3K; ↓ AKT; ↓ mTOR; ↓ P70S6K mRNA	[50]
Shikonin M12	Colorectal	Intrinsic	↑ ROS; ↓ MMP	[100]
β, β-Dimethylacrylshikonin	Colorectal (HCT-116)	Intrinsic	↓ Bcl-2; ↑ Bax	[43]
	Skin (BRAF/NRAS-mutated)	Intrinsic	↑ NOXA; ↑ Caspase-3 activation	[102]
	Chordoma (MUG-Chor1, U-CH2)	Intrinsic	↑ NOXA; ↑ PUMA	[103]
Cyclopropyl-acetylshikonin	Skin (WM9, WM164)	Intrinsic	↑ Caspase-3/7	[98]
Acetylshikonin and cyclopropylshikonin	Chondrosarcoma (Cal 78, SW-1353)	Intrinsic	↑ Caspase-7/9; ↑ NOXA; ↑ γH2AX	[68]
E2	TNBC (MDA-MB-231)	PDK1/PDHC axis	↑ ROS; ↑ Bax; ↑ Fas	[99]

**Abbreviations:** ROS: reactive oxygen species; MMP: mitochondrial membrane potential; Bcl-2: B-cell lymphoma 2; Bax: Bcl-2-associated X protein; NOXA and PUMA: pro-apoptotic proteins; PARP: poly (ADP-ribose) polymerase; TNBC: triple-negative breast cancer; PDK1: pyruvate dehydrogenase kinase 1; PDHC: pyruvate dehydrogenase complex; γH2AX: DNA damage marker; ↑: up-regulated genes; ↓: downregulated genes.

**Table 3:** Synergistic outcomes of shikonin in cancer therapy.

Cancer Type	Combination	IC_50_ (μM) Reduction	Apoptosis (% Annexin V+)	Key Molecular Changes	Ref.
Lung (A549, PC9)	Shikonin + Cisplatin	A549 (5.74 µM); PC9 (6.30 µM)	A549 (+650%); PC9 (+525%)	↓ PKM2; ↓ Glycolysis; ↓ Exosome	[141]
Ovary (A2780/DDP, SKOV3/DDP, OVCAR4/DDP)	Shikonin + Cisplatin	A2780/DDP (23.46 µM); SKOV3/DDP (50.06 µM); OVCAR4/DDP (18.06 µM)	Not reported	↑ HMOX1; ↑ Heme breakdown; ↑ Fe^2+^; ↑ ROS; ↑ Lipid peroxidation; ↑ Ferroptosis	[147,149]
Oesophagus (KYSE-150, KYSE-270)	Shikonin + Paclitaxel	Not reported	KYSE270 (+ ~367%)	↑ p53 activation; ↓ Bcl-2 expression	[154]
Pancreas (PANC-1, BxPC-3)	Shikonin + Gemcitabine	PANC-1 (1.800 μM); BxPC-3 (3.18 μM)	PANC-1 (3 μM—14.66%; 5 μM—83.35%; 10 μM—90.50%); BxPC-3: (3 μM—~7%; 5 μM—~14%; 10 μM—~17%)	↓ PAK1; ↓ Downstream signalling; ↑ Apoptosis	[161]
Breast (MDA-MB-435, MCF-7)	Shikonin + 4-hydroxytamoxifen	Not reported	MDA-MB-435S (26.3%); MCF-7( 22.9%)	↑ ROS; ↓ MMP; ↑ Apoptosis	[166]
Breast (MCF-7R)	Shikonin + Tamoxifen	Not reported	Not reported	↑ lncRNA uc.57; ↓ BCL11A; ↓PI3K/AKT and MAPK pathways	[170]
Breast (MDA-MB-468)	Shikonin + anti-PD-1	3.59 μM	Z-VAD-FMK (~2%); Nec-1 (~39%)	↑ RIP1K and RIP3K; ↑ ROS; ↓ MMP; Necroptosis	[177]
Colon (CT26)	Shikonin + anti-PD-1	Not reported	12.47% (5 μM); 20.17% (10 μM)	↑ Calreticulin exposure; ↑ Hsp70; APCs activation	[181]
Colon (HCT116, LN428, H460, A549)	β, β-Dimethylacrylshikonin + IR	Not reported	~200%	↑ ROS; ↑ DNA damage; ↑ Apoptosis	[150]
Kidney (SKRC-17, RCC-53)	Shikonin + Ipilimumab	1.32 μM	~344%	↓ FoxP3^+^ Tregs; ↑ Activation of CD8^+^ and CD4^+^ T cells	[185]

**Abbreviations:** Bcl-2: B-cell lymphoma 2; ROS: reactive oxygen species; MMP: mitochondrial membrane potential; lncRNAs: long non-coding RNAs; MAPK: mitogen-activated protein kinase; RIPK1: receptor-interacting protein kinase1; RIPK3: receptor-interacting protein kinase3; Tregs: regulatory T cells; ↑: increased; ↓: decreased.

**Table 4:** Preclinical and clinical evaluation of shikonin and its derivatives in cancer models.

Cancer Type/Model	Agent	Mechanism of Action	Main Outcomes	Ref.
Melanoma (A375 xenograft, mice)	Shikonin (5.0 mg/kg)	↑ Apoptotic proteins	Greater tumour suppression vs. dacarbazine	[111]
Melanoma (B16F10 lung metastasis, mice)	Shikonin + Quercetin	PKM2 inhibition, ↓ platelet–tumour interaction, ↓ glycolysis	Fewer metastatic nodules; no adverse effects	[186]
Breast cancer (Orthotopic; TNBC, MCF-7)	Shikonin	PDK1 inhibition, EMT suppression	↓ Tumour invasion and growth; TNBC sensitivity	[17,187]
Breast cancer (MCF-7 xenograft, mice)	Shikonin + siRNA/TGF-β nanoparticle	ICD induction (calreticulin exposure), improved biodistribution	21.8% ICD; tumour-specific targeting	[188]
HNSCC (xenograft, mice)	Shikonin	Suppression of FAM83A/PKM2 axis	↓ PKM2 expression; ↓ tumour progression	[189]
Cervical and ovarian cancer (xenograft)	Shikonin	Apoptosis, anti-metastatic activity	↓ Tumour growth; ↑ cisplatin sensitivity	[17,147]
Oral cancer (mouse model)	Shikonin	Apoptosis induction	Tumour growth inhibition	[190]
Pancreatic cancer (cell models)	β-hydroxyisovaleryl-shikonin	↑ ROS, apoptosis	Enhanced cytotoxicity	[154]
Clinical trial (lung cancer, 1991)	Shikonin (dose unclear)	Not reported	>25% tumour reduction; mean survival ~10 months	[191]

**Abbreviations:** PKM2: pyruvate kinase isoform M2; PDK1: pyruvate dehydrogenase kinase 1; EMT: epithelial–mesenchymal transition; TNBC: triple negative breast cancer; ICD: immunogenic cell death; ROS: reactive oxygen species ↑: increased; ↓: decreased.

## Data Availability

No new data were created or analysed in this study.

## Abbreviations

The following abbreviations are used in this manuscript:

1-κB⍺	I kappaB alpha
4-OHT	4-hydroxytamoxifen
5-FU	5-fluorouracil
AMPK	AMP-activated protein kinase
APCs	Antigen-presenting cells
ATF4	Activating transcription factor 4
β-carotene	Beta-carotene
Bax	Bcl-2-associated X protein
Bcl	B-cell lymphoma
Bid	BH3-interacting domain death agonist
Ca^2+^	Calcium
ccRCC	Clear cell renal cell carcinoma
CDK	Cyclin-dependent kinase
CHOP	C/EBP homologous protein
CML	Chronic myeloid leukaemia
CRC	Colorectal cancer
CRT	Calreticulin
CSCs	Cancer stem cells
CTL	Cytotoxic T lymphocytes
DAMPs	Damage-associated molecular patterns
DC	Dendritic cells
DISC	Death-inducing signalling complex
DNases	Deoxyribonucleases
DR	Death receptor
DX	Doxorubicin
EGFR	Epidermal growth factor receptor
EMT	Epithelial–mesenchymal transition
ER	Endoplasmic reticulum
ER^+^	Oestrogen receptor–positive
ER^−^	Oestrogen receptor–negative
ERK	Extracellular signal-regulated kinase
ESCC	Oesophageal squamous cell carcinoma
ESM1	Endothelial cell specific molecule 1
FADD	Fas-associated death domain
FASL	Fas ligand
Fe^2+^	Ferrous iron
FPE	First-pass effect
GC	Gastric cancer
GOT-1	Glutamic-oxaloacetic transaminase 1
GPER	G protein-coupled oestrogen receptor
GRP78	Glucose-regulated protein 78
GSDME	Gasdermin E
GTPBP4	Guanosine triphosphate binding protein 4
HCC	Hepatocellular carcinoma
HDAC	Histone deacetylase
HIF-1α	Hypoxia-inducible factor 1-alpha
HMOX1	Heme oxygenase 1
HNSCC	Head and neck squamous cell carcinomas
HPLC	High-performance liquid chromatography
HSCCC	High-speed counter-current chromatography
ICD	Immunogenic cell death
IHC	Immunohistochemistry
IR	Ionising radiation
IV	Intravenous
JNK	c-Jun N-terminal kinase
KM	Kunming
KRAS	Kirsten rat sarcoma virus
lncRNAs	Long non-coding RNAs
MAE	Microwave-assisted extraction
MAPK	Mitogen-activated protein kinase
MitoROS	Mitochondrial ROS
MLKL	Mixed lineage kinase domain-like protein
MM	Multiple myeloma
MMP	Mitochondrial membrane potential
MMP-2	Matrix metalloproteinase-2
MMP-9	Matrix metalloproteinase-9
MS	Murashige and Skoog
NF-κB	Nuclear factor kappa B
NHE1	Sodium–hydrogen exchanger 1
NSCLC	Non-small cell lung cancer
OSCC	Oral squamous cell carcinoma
OXA	Oxaliplatin
PAK1	p21-activated kinase 1
PAMPs	Pathogen-associated molecular patterns
PARP	Poly (ADP-ribose) polymerase
PBMC	Patient peripheral blood mononuclear cell
PCD	Programmed cell death
PDK1	Pyruvate dehydrogenase kinase 1
PFKFB2	6-phosphofruto-2-kinase/fructose-2,6-biphosphate
PHD3	Prolyl hydroxylase 3
PKM2	Pyruvate kinase isoform M2
PTEN	Phosphatase and tensin homologue
PUMA	p53 upregulated modulator of apoptosis
PYCR1	Pyrroline-5-carboxylate reductase 1
rhApo2L/TRAIL	Apo2 ligand/tumour necrosis factor-related apoptosis-inducing ligand
RIPK1	Receptor-interacting protein kinase1
RIPK3	Receptor-interacting protein kinase3
ROS	Reactive oxygen species
RT-PCR	Real-time polymerase chain reaction
SC-CO_2_	Supercritical carbon dioxide
SCLC	Small cell lung cancer
SD	Sprague Dawley
STP	Sarcoma-targeting-peptide
TAM	Tamoxifen
TCM	Traditional Chinese Medicine
TGF-β	Transforming growth factor β
TIME	Tumour immune microenvironment
TLRs	Toll-like receptors
TME	Tumour microenvironment
TNBC	Triple negative breast cancer
TNFα	Tumour necrosis factor-α
TRAP1	Tumour necrosis factor receptor-associated protein 1
Tregs	Regulatory T Cells
TrxR1	Thioredoxin reductase 1
UAE	Ultrasonic-assisted extraction
XBP-1	X-box binding protein 1
Zn-SHK-PEG	Zinc-shikonin-polyethylene glycol
